# Evaluating short-term corneal endothelial alterations post-intravitreal Anti-VEGF injections in treatment naïve eyes

**DOI:** 10.22336/rjo.2025.34

**Published:** 2025

**Authors:** Anandsagar Kanna, Avadhesh Oli, Santosh Kumar, Bv Rao, Mohan S, Simran Dhami

**Affiliations:** Department of Ophthalmology, Command Hospital Airforce, Bangalore, Karnataka, India

**Keywords:** corneal endothelium, specular microscopy, endothelial cell density, coefficient of variation, anti-VEGF injections, CCT = Central Corneal Thickness, CV = Coefficient of Variation, ECD = Endothelial Cell Density, OCT = Optical Coherence Tomography, SP = Specular Microscopy, VEGF = Vascular Endothelial Growth Factors

## Abstract

**Background and objective:**

The cornea is the outermost transparent layer of the eye. Various anatomical and physiological factors, such as a healthy functioning monolayer of corneal endothelial cells, play an essential role in maintaining corneal transparency. Conditions or events that cause endothelial loss beyond the threshold result in loss of corneal transparency. The present study aimed to evaluate the short-term effects of intravitreal anti-VEGF injections on the corneal endothelium using non-contact specular microscopy in patients undergoing anti-VEGF injections for various retinal diseases.

**Materials and methods:**

This prospective cohort study included 47 eyes of 47 treatment-naïve patients who received intravitreal anti-VEGF injections for various retinal diseases. Using a non-contact specular microscope, pre-injection parameters, including ECD, cell count, CV, and hexagonality, were compared with those on post-injection days 1, 1st week, 1st month, and 3rd month. A statistically significant result was defined as a “p” value of less than 0.05 using the appropriate test of significance.

**Results:**

Early morphological changes in endothelial cells were indicated by a significant increase in the mean value of the coefficient of variation of the area of endothelial cells in the first week (p < 0.001) and the first month (p < 0.027) of the post-injection period. However, at the follow-up examination 30 days later, no noticeable change in the patient’s ECD, cell count, or hexagonal shape could be detected under the specular microscope.

**Discussion:**

This prospective cohort study evaluated the effects of intravitreal anti-VEGF injections on corneal endothelial morphology in 47 treatment-naïve, non-diabetic patients with various retinal conditions. While endothelial cell count, density, and hexagonality remained stable, a transient increase in polymegathism was observed at both day 7- and one-month post-injection. No differences were found between phakic and pseudophakic eyes or between different anti-VEGF agents (Aflibercept vs. Ranibizumab).

**Conclusion:**

Intravitreal anti-VEGF injections were found to induce morphological alterations in corneal endothelial cells during the first week and first month following injection, as evidenced by an increase in the coefficient of variation. However, these endothelial changes are subtle and do not typically translate into clinical concerns.

## Introduction

Light is transmitted to the retina by the cornea, a densely innervated, transparent tissue [1-3]. Cells produce a signal protein called vascular endothelial growth factor (VEGF), which promotes the development of blood vessels [4]. Anti-VEGFs, such as ranibizumab and bevacizumab, have been shown to have higher levels in the aqueous humour following intravitreal injections in animal models, even though the pharmacokinetic profile of these injections in human eyes has not yet been conclusively established. Research has indicated that the corneal endothelium has VEGF receptors. Thus, following intravitreal injections, ranibizumab in the aqueous humour may have an impact on corneal endothelium function [5].

Intravitreal anti-VEGF injections are the cornerstone of treating various retinal vascular diseases, as their effectiveness has been proven beyond doubt [6,7]. Although the safety of intravitreal anti-VEGF injections has been established, the possibility of corneal decompensation due to unfavourable effects on the corneal endothelium remains, which has been investigated in animals and human corneal endothelial cells cultured by different authors [8]. Research conducted on animals that received intracameral injections of anti-VEGFs has suggested several mechanisms for endothelial cell damage, including oxidative stress-induced apoptosis of corneal endothelial cells [9], as well as modifications to the nerve growth factor (NGF) pathway resulting in the overexpression of cCasp3, which causes apoptosis and a decrease in endothelial cells [10]. Studies have revealed increased hexagonality and electron microscopic changes in the morphology of the corneal endothelium resulting from the formation of antibodies against anti-VEGF [11]. Nevertheless, there is less endothelial cell loss than after glaucoma or cataract surgery [12].

This study was necessary because many patients requiring intravitreal anti-VEGF injections have diabetes of advanced age, have physiologically low endothelial cell counts, and often require multiple injections, leaving their corneal endothelium vulnerable [13,14]. To sustain corneal deturgescence and prevent corneal edema, the endothelial cell density must remain above a threshold level [15,16]. Therefore, understanding how intravitreal anti-VEGF injections affect corneal endothelial parameters is crucial for preventing corneal complications, such as corneal decompensation.

Our study is notable in that most patients in previous studies on this topic had diabetes. The inclusion of diabetic patients may have altered the results of these studies and led to biased conclusions, as diabetes is known to affect corneal endothelium parameters independently. Diabetes was excluded as a criterion in our study, allowing us to eliminate this confounding bias.

## Materials and methods

After obtaining approval and clearance from the institutional ethics committee, a total of 47 eyes from 47 patients who fulfilled the inclusion criteria for intravitreal anti-VEGF injections were enrolled in the study, following informed written consent.

Data was collected about age, gender, occupation, presence of concomitant systemic diseases, topical and systemic medications, and any history of ocular surgery. BCVA, IOP, slit lamp examination of anterior segment followed by dilated fundus examination at every visit. Patients were divided into two groups. Group 1 included patients who received Ranibizumab injections, and Group 2 included patients who received Aflibercept injections. At baseline, before intravitreal anti-VEGF injections, the corneal endothelium was assessed using a non-contact specular microscope (Tomey EM-4000, Tomey USA, Arizona, USA). Patients were always asked to look at the central fixation target, and the auto-alignment function was used. An average of 3 readings was taken. Endothelial cell density (ECD) was calculated using the instrument’s built-in software.

All four pre-injections endothelial parameters which included cell density (number of cells per mm^2^), cell number (number of cells analysed in the frame), coefficient of variation (standard deviation of mean cell area divided by mean cell area, an indicator of polymegathism or change in the size of cells) and hexagonality (percentage of hexagonal cells in the endothelial mosaic, an indicator of polymorphism or change in the shape of cells) were compared on day 1, 1^st^ week, 1^st^ month and 3^rd^ month of post injection.

### Inclusion criteria

Treatment-naïve patients who received only one intravitreal anti-VEGF injection for various retinal diseases within the past 3 months.

### Exclusion Criteria

Patients with a history of acute ocular infections, ocular trauma, previous intravitreal anti-VEGF injections, corneal diseases (acquired or genetic), and those with a history of diabetes mellitus were excluded from the study.

### Statistical analysis

Descriptive and inferential statistical analysis was carried out. Results on continuous measurements were presented as Mean ± SD (Min-Max), and results on categorical measurements were presented in Number (%). Significance was assessed at a 5% level of significance. Student t test (two-tailed, independent) was used to find the significance of study parameters on a continuous scale between two groups (intergroup analysis) on metric parameters. Levene’s test for homogeneity of variance was performed to assess the homogeneity of variance. The Statistical software SPSS 22.0 and R environment version 3.2.2 were used for data analysis, while Microsoft Word and Excel were utilized to generate graphs, tables, and other visual aids.

## Results

The study included 47 eyes from 47 treatment-naïve patients who underwent intravitreal injections of anti-VEGF agents (ranibizumab or aflibercept). Thirty-two were men and 15 were women with a mean age of 60.87 years (range 31-84 years). 40 (81.5%) were phakic and 7 (14.9%) were pseudophakic, respectively. The patients had received anti-VEGF injections for indications such as retinal vein occlusion (RVO) in 21 patients (44.6%), choroidal neovascular membrane (CNVM) in 15 patients (31.9%), and pseudophakic cystoid macular edema (PCME) in 7 patients (14.89%). All 47 patients received a single intravitreal anti-VEGF injection. Thirty-one of them (66.6%) received an injection of aflibercept, and 16 of them (34%) received ranibizumab (**Table 1**). All patients completed the 3-month follow-up. No systemic or ocular adverse events were recorded during this period.

**Table 1:** Baseline characteristics of patients

Characteristics	Patients (N-47)	
Age in Mean (Range)	60.87 years (31-84)	
Gender	Male	32 (68.1%)
	Female	15 (31.9%)
Laterality	Right	26 (55.3%)
	Left	21 (44.7%)
Diagnosis	RVO	21 (44.6)
	CNVM	15 (31.9)
	PCME	7 (14.8)
Drug	Aflibercept	16 (34%)
	Ranibizumab	31 (66%)
Lens status	Phakic	40 (85.1%)
	Pseudophakic	7 (14.9%)

RVO = Retinal vein occlusion, CNVM = Choroidal neovascular membrane, PCME = Pseudophakic cystoid macular oedema

The mean pre-injection endothelial parameters of all 47 patients were as follows: cell density, 2653.72 ± 250.3; cell count, 258.02 ± 32.07; coefficient of variation, 35.55 ± 3.82; and hexagonality, 48.7 ± 6.66. There was a statistically significant change in the morphology of endothelial cells, as evidenced by a change in the coefficient of variation (p < 0.001 and 0.027 at the end of 1 week and 1 month, respectively). No significant changes in endothelial cell density, cell number, and hexagonality were observed (**Table 2**).

**Table 2:** ECD, CC, CV, and Hex comparison before and after injections in all eyes used in this study

		Min-Max	Mean ± SD	p value
Endothelial cell density	Pre-injection	1918-3268	2653.72 ± 250.3	-
	Day 1	1966-3224	2659.81 ± 247.38	0.652
	1st Week	2072-3160	2640.19 ± 247.47	0.292
	1^st^ Month	1926-3197	2646.96 ± 246.34	0.587
	3^rd^ Month	1936-3208	2659.55 ± 252.37	0.655
Cell count	Pre-injection	177-357	258.02 ± 32.07	-
	Day 1	145-331	261.57 ± 34.11	0.399
	1st Week	147-321	257.83 ± 36.6	0.958
	1^st^ Month	202-347	258.13 ± 27.43	0.976
	3^rd^ Month	181-346	263.51 ± 32.1	0.012*
Coefficient of variation	Pre-injection	25-43	35.55 ± 3.82	-
	Day 1	26-47	36.34 ± 4.63	0.053+
	1st Week	26-46	36.79 ± 4.18	<0.001**
	1^st^ Month	27-50	36.74 ± 4.61	0.027*
	3^rd^ Month	26-47	35.83 ± 3.94	0.352
Hexagonality	Pre-injection	37-70	48.7 ± 6.66	-
	Day 1	36-73	48.53 ± 7.38	0.840
	1st Week	38-73	47.55 ± 7.32	0.133
	1^st^ Month	34-70	47.64 ± 6.81	0.128
	3^rd^ Month	35-70	47.36 ± 7.08	0.078+

The measurements obtained at all time points were compared between the two groups: one receiving aflibercept injections and the other receiving ranibizumab injections. The results are presented in **Table 3**. Of all the evaluated parameters, no statistically significant changes were noted from the baseline to 3 months post-injection.

**Table 3:** ECD, CC, CV, and Hex comparison between the Ranibizumab and Aflibercept groups

		INTRAVITREAL INJECTION TYPE		Total	p Value
		RANIBIZUMAB	AFLIBERCEPT		
Endothelial cell density	Pre-injection	2690.44 ± 244.98	2634.77 ± 254.89	2653.72 ± 250.3	0.476
	Day 1	2688.06 ± 239.29	2645.23 ± 254.09	2659.81 ± 247.38	0.579
	1st Week	2675.38 ± 254.87	2622.03 ± 245.82	2640.19 ± 247.47	0.490
	1^st^ Month	2642.69 ± 251.3	2649.16 ± 247.91	2646.96 ± 246.34	0.933
	3^rd^ Month	2674.06 ± 215.61	2652.06 ± 272.48	2659.55 ± 252.37	0.781
Cell count	Pre-injection	257.63 ± 32.16	258.23 ± 32.55	258.02 ± 32.07	0.952
	Day 1	268.13 ± 27.41	258.19 ± 37.07	261.57 ± 34.11	0.350
	1st Week	261.69 ± 40.01	255.84 ± 35.24	257.83 ± 36.6	0.609
	1^st^ Month	260 ± 25.1	257.16 ± 28.92	258.13 ± 27.43	0.741
	3^rd^ Month	263.19 ± 31.96	263.68 ± 32.69	263.51 ± 32.1	0.961
Coefficient of variation	Pre-injection	35.25 ± 2.41	35.71 ± 4.41	35.55 ± 3.82	0.700
	Day 1	35.81 ± 2.81	36.61 ± 5.36	36.34 ± 4.63	0.580
	1st Week	36.38 ± 3.88	37 ± 4.37	36.79 ± 4.18	0.632
	1^st^ Month	36.38 ± 5.21	36.94 ± 4.34	36.74 ± 4.61	0.697
	3^rd^ Month	35.94 ± 3.26	35.77 ± 4.3	35.83 ± 3.94	0.895
Hexagonality	Pre-injection	48.38 ± 5.57	48.87 ± 7.24	48.7 ± 6.66	0.812
	Day 1	46.94 ± 4.54	49.35 ± 8.44	48.53 ± 7.38	0.292
	1st Week	46.56 ± 4.76	48.06 ± 8.37	47.55 ± 7.32	0.511
	1^st^ Month	47.88 ± 3.95	47.52 ± 7.95	47.64 ± 6.81	0.866
	3^rd^ Month	46.31 ± 5.12	47.9 ± 7.92	47.36 ± 7.08	0.471

**Fig. 1:**
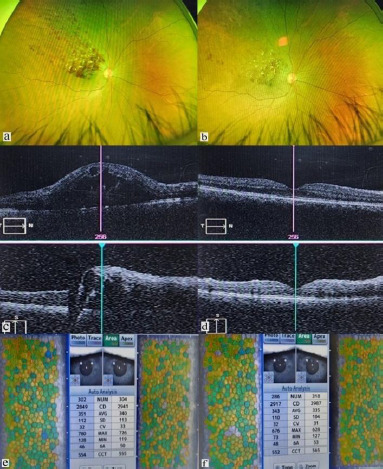
Wide field fundus, OCT, and specular microscopic images of a 54-year-old male case of BRVO right eye before and after 3 months of Ranibizumab injection

**Fig. 2:**
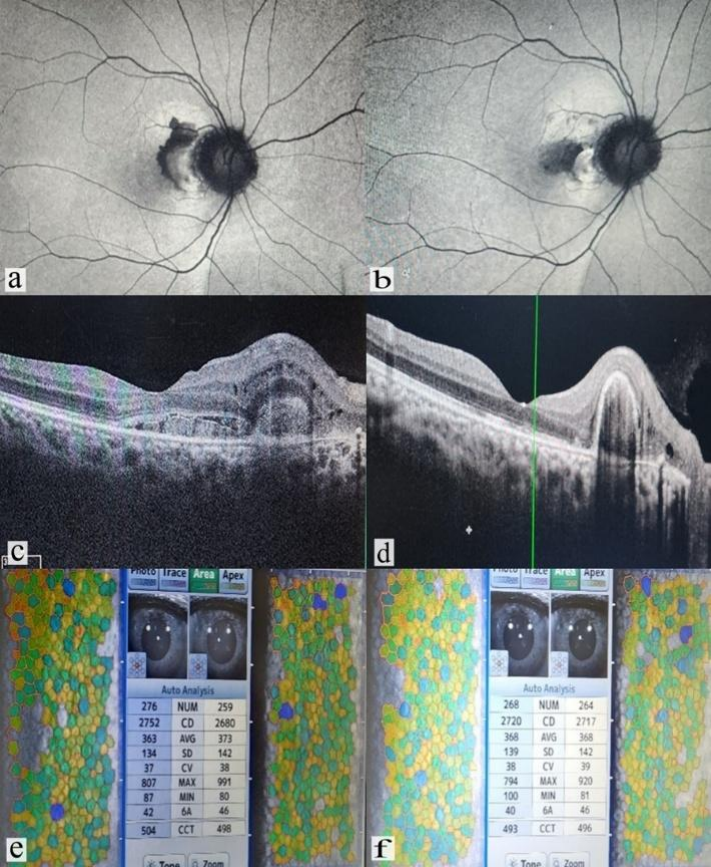
Wide field fundus, OCT, and specular microscopic images of a 31-year-old female case of CNVM right eye before and after 3 months of Aflibercept injection

As a representative case, the base line corneal parameters of specular microscopy, wide field fundus images and optical coherence tomography (OCT) images of patient with branch retinal vein occlusion (BRVO) and choroidal neovascular membrane (CNVM) before and after intravitreal anti-VEGF injections are shown in **Fig. 1 and 2** with significant anatomical and functional improvement.

## Discussion

In our prospective cohort study, anti-VEGF intravitreal injections were administered to the eyes of 47 treatment-naïve patients for various retinal conditions. Since diabetes mellitus itself may be linked to substantial alterations in the corneal endothelium, diabetic patients were excluded from the study. In the present investigation, we observed a notable shift in cell morphology, as evidenced by changes in the coefficient of variation, suggestive of polymegathism (increased endothelial cell size) on the seventh day and at the one-month follow-up. The cell count, density, and hexagonality did not, however, exhibit any appreciable changes. There were no notable differences in endothelial parameters between the phakic and pseudophakic patient groups when they were compared.

A significant number of studies conducted by various authors have found no changes in the corneal endothelium up to the 6th month post-injection, even with multiple anti-VEGF injections [17-19]. However, few other studies have found that the use of intravitreal anti-VEGF is associated with changes in anterior chamber depth (ACD) and endothelial cell density (ECD) [20] and significant changes in cell size on specular microscopy [21]. Another author detected morphological changes, such as a decrease in the hexagonal shape of endothelial cells [22]. However, a common occurrence in these studies was that the majority of patients undergoing intravitreal anti-VEGF injections were treated for diabetic retinopathy; hence, these changes could be attributed to the endothelial changes associated with diabetes itself, rather than to the direct effects of anti-VEGF on the corneal endothelium.

The toxic effects of anti-VEGF injections on the corneal endothelial cells have been documented by various studies done on animals and cultured endothelial cells. Although the exact mechanism by which anti-VEGF injections induce changes in the corneal endothelium is not well understood, a few authors have proposed different mechanisms. One study suggested that the toxic effects of anti-VEGF (Bevacizumab) are due to the development of anti-bevacizumab antibodies in cultured human corneal endothelium; however, the concentration of bevacizumab used was higher than that typically used in clinical scenarios [8]. Another study done on rat’s eyes undergoing intracameral injections of aflibercept and ranibizumab, found the reduction in cell count may be due to endothelial apoptosis mediated by changes in nerve growth factor (NGF) pathway leading to changes in the expression of NGF precursor (proNGF) and p75 neurotrophin receptors (p75NTR) and increased expression of cleaved-caspases 3 (c-Casp3) [9]. A few more authors attributed the toxic effects of intracameral bevacizumab on albino rats to oxidative stress on corneal endothelium, leading to apoptosis [10]. In a recent study on mice receiving intracameral injections of ranibizumab and bevacizumab, electron microscopic changes in the corneal endothelium were observed. They proposed that the loss of the hexagonal shape of the corneal endothelium was due to the increased intraocular pressure following injections, which led to the loss of intercellular junctions and a reduction in microvilli [11].

Since the introduction of intravitreal anti-VEGF injections for various retinal conditions such as diabetic retinopathy, retinal vein occlusion, and neovascular age-related macular degeneration, these injections have become the first choice of treatment, replacing other conventional therapies like laser. The rise in the number of anti-VEGF injections is not without proven side effects, such as a transient increase in intraocular pressure or a risk of post-operative infections, including endophthalmitis. Most of the patients undergoing these treatments are elderly with age-related reduction in corneal endothelial count, which might make it even worse if they have coexisting diabetes or a history of complicated previous ocular surgeries, as these factors are known to reduce the endothelial cells further. The repeated injections of these anti-VEGF agents may harm the functioning of the endothelial cells, as the interval between injections may not be sufficient for the cells to recover to normal before the next injection is administered. Hence, it is essential to rule out any significant effects of these injections on the corneal endothelial cells, as these cells are crucial for maintaining the cornea’s transparency.

In our study, we did not find any significant difference in the change in corneal parameters based on the type of anti-VEGF used (Aflibercept vs. Ranibizumab); however, a longer follow-up is required to draw any clinical conclusions.

### Limitations of this study

Our study had a short follow-up period with only one point of exposure to intravitreal anti-VEGF injection. Hence, further investigations in this area, with a larger sample size and a longer follow-up duration, are recommended to establish the relationship between the use of intravitreal anti-VEGF injections and their effects on the corneal endothelium. However, it is recommended to take precautions in elderly patients with risk factors such as diabetes, a history of intraocular surgeries, and in patients with pseudoexfoliation (PEX).

## Conclusion

Intravitreal anti-VEGF injections have been reported to induce increased coefficient of variation and other morphological changes in corneal endothelial cells from the first post-injection period to one month after injection. According to our research, these injections typically result in slight, temporary, and clinically insignificant changes to the morphology of the corneal endothelium cells, despite some evidence to the contrary.

## References

[1] Sridhar MS (2018). Anatomy of cornea and ocular surface. Indian J Ophthalmol.

[2] DelMonte DW, Kim T (2011). Anatomy and physiology of the cornea. J Cataract Refract Surg.

[3] Edelhauser HF (2006). The balance between corneal transparency and edema: the Proctor Lecture. Invest Ophthalmol Vis Sci.

[4] Shibuya M (2011). Vascular Endothelial Growth Factor (VEGF) and Its Receptor (VEGFR) Signaling in Angiogenesis: A Crucial Target for Anti-and Pro-Angiogenic Therapies. Genes Cancer.

[5] Bakri SJ, Snyder MR, Reid JM, Pulido JS, Ezzat MK, Singh RJ (2007). Pharmacokinetics of intravitreal ranibizumab (Lucentis). Ophthalmology.

[6] Boyer DS, Hopkins JJ, Sorof J, Ehrlich JS (2013). Anti-vascular endothelial growth factor therapy for diabetic macular edema. Ther Adv Endocrinol Metab.

[7] Blinder KJ, Dugel PU, Chen S, Jumper JM, Walt JG, Hollander DA, Scott LC (2017). Anti-VEGF treatment of diabetic macular edema in clinical practice: effectiveness and patterns of use (ECHO Study Report 1). Clin Ophthalmol.

[8] Yoeruek E, Spitzer MS, Tatar O, Aisenbrey S, Bartz-Schmidt KU, Szurman P (2007). Safety profile of bevacizumab on cultured human corneal cells. Cornea.

[9] Gharbiya M, Bruscolini A, Sacchetti M, Rosso P, Carito V, Segatto M, Fico E, Tirassa P, Lambiase A (2018). In vivo antivascular endothelial growth factor treatment induces corneal endothelium apoptosis in rabbits through changes in p75NTR-proNGF pathway. J Cell Physiol.

[10] Akal A, Ulas T, Goncu T, Guldur ME, Kocarslan S, Taskin A, Sezen H, Ozkan K, Yilmaz OF, Buyukhatipoglu H (2015). Evaluating the safety of intracameral bevacizumab application using oxidative stress and apoptotic parameters in corneal tissue. Int J Ophthalmol.

[11] Ari S, Nergiz Y, Aksit I, Sahin A, Cingu K, Caca I (2015). Evaluation of intracameral injection of ranibizumab and bevacizumab on the corneal endothelium by scanning electron microscopy. J Ocul Pharmacol Ther.

[12] Bourne RR, Minassian DC, Dart JK, Rosen P, Kaushal S, Wingate N (2004). Effect of cataract surgery on the corneal endothelium: modern phacoemulsification compared with extracapsular cataract surgery. Ophthalmology.

[13] Islam QU, Saeed MK, Mehboob MA (2017). Age related changes in corneal morphological characteristics of healthy Pakistani eyes. Saudi J Ophthalmol.

[14] Shenoy R, Khandekar R, Bialasiewicz A, Al Muniri A (2009). Corneal endothelium in patients with diabetes mellitus: a historical cohort study. Eur J Ophthalmol.

[15] Chaurasia S, Vanathi M (2021). Specular microscopy in clinical practice. Indian J Ophthalmol.

[16] McCarey BE, Edelhauser HF, Lynn MJ (2008). Review of corneal endothelial specular microscopy for FDA clinical trials of refractive procedures, surgical devices, and new intraocular drugs and solutions. Cornea.

[17] Joshi M, Naik MP, Sarkar L (2019). Effect of intravitreal anti-vascular endothelial growth factor on corneal endothelial cell count and central corneal thickness in Indian population. J Family Med Prim Care.

[18] Ulutas HG, Yener NP (2021). Effects of Intravitreal Injection on Ocular Surface and Anterior Segment Parameters. Beyoglu Eye J.

[19] Lass JH, Benetz BA, Menegay HJ, Tsipis CP, Cook JC, Boyer DS, Singer M, Erickson K, Saroj N, Vitti R, Chu KW, Moini H, Soo Y, Cheng Y (2018). Effects of Repeated Intravitreal Aflibercept Injection on the Corneal Endothelium in Patients With Age-Related Macular Degeneration: Outcomes From the RE-VIEW Study. Cornea.

[20] Arslan GD, Guven D, Alkan AA, Kacar H, Demir M (2019). Short term effects of intravitreal anti-vascular endothelial growth factor agents on cornea, anterior chamber, and intraocular pressure. Cutan Ocul Toxicol.

[21] Muto T, Machida S (2019). Effect of intravitreal aflibercept on corneal endothelial cells: a 6-month follow-up study. Clin Ophthalmol.

[22] Qi Y, Cui L, Zhang L, Yan C, Jiang Y, Ye S, Ji L, Qiu Y, Zhang L (2023). Effect of repeated intravitreal anti-vascular endothelial growth factor drugs on corneal nerves. Medicine (Baltimore).

